# Platelet-derived sCD40L: specific inflammatory marker for early-stage severe acute respiratory syndrome coronavirus 2 infection

**DOI:** 10.1186/s12985-021-01680-3

**Published:** 2021-10-29

**Authors:** Hind Hamzeh-Cognasse, Alexandre Mansour, Florian Reizine, Patrick Mismetti, Isabelle Gouin-Thibault, Fabrice Cognasse

**Affiliations:** 1 INSERM, U1059, SAINBIOSE, Université de Lyon, Université Jean-Monnet, F-42023 Saint-Etienne, France; 2 Department of Anesthesiology Critical Care Medicine and Perioperative Medicine, CHU de Rennes, F-35000 Rennes, France; 3 INSERM, CIC 1414, Université de Rennes, CHU de Rennes, F-35000 Rennes, France; 4 Intensive Care Unit, CHU de Rennes, F-35000 Rennes, France; 5 Department of Vascular Medicine and Therapeutics, INNOVTE, CHU de St-Etienne, F-42055 Saint-Etienne, France; 6 INSERM, CIC-1408, CHU de Saint-Etienne, F-42055 Saint-Etienne, France; 7 Department of Biological Hematology, CHU de Rennes, F-35000 Rennes, France; 8 Etablissement Français du Sang Auvergne-Rhône-Alpes, 25 Boulevard Pasteur, F-42100 Saint-Etienne, France

**Keywords:** Platelets, Innate immunity, CD40L, Inflammation, SARS-CoV2

## Abstract

**Background:**

The SARS-CoV-2 virus is the causing agent of the Coronavirus disease 2019 (COVID-19) characterized by a huge pro-inflammatory response and coagulation disorders that may lead to for its severe forms, in organ failure or even death. As major players of thrombo-inflammation, platelets release large amounts of immunomodulatory molecules and regulate leukocyte and endothelial activity, which are both altered in COVID-19. Altogether, this makes platelets a very likely actor of the thrombo-inflammatory complications of COVID-19. Thus, we propose to identify a platelet inflammatory signature of severe COVID-19 specifically modulated throughout the course of the disease.

**Methods:**

Luminex technology and enzyme-linked immunosorbent assay were used to assess plasma levels of platelet inflammatory markers in patients with severe acute respiratory syndrome coronavirus 2 infection on admission and for 14 days afterwards.

**Results:**

In accordance with the observations of other teams, we evidence that the plasma levels of the platelet soluble (s)CD40L is significantly elevated in the early stages of the disease. Interestingly we observe that the plasma level of sCD40L decreases overtime while that of sCD62P increases significantly.

**Conclusions:**

Our data suggest that there is a platelet signature of inflammatory response to SARS-COv-2 infection which varies overtime and could serve as monitoring biomarkers of patient inflammatory state.

*Clinical trial registration number*: 2020-A01100-39; title: Human Ab Response & immunoMONItoring of COVID-19 Patients, registration date: 05/25/2020; URL of the registry: https://clinicaltrials.gov/ct2/history/NCT04373200?V_5=View.

## Background

Beyond their contribution to hemostatic response, platelets behave as immune cells owing to their innate immunity receptors, including toll-like receptors, enabling them to sense danger signals, as neutrophils, macrophages, or dendritic cells do [1]. Upon activation, platelets release immune mediators and chemokines such as soluble (s)CD40L, sCD62P, or CXCL4 from their granules or membranes [1]. sCD40L and sC62P from platelets mediate thrombotic and inflammatory processes, contributing to inflammation associated to viral infection and increased cardiovascular disease risk [2]. In patients with coronavirus disease (COVID-19), platelet hyperreactivity [3] and upregulated release of soluble immunomodulatory factors [4] have been described, which suggests a platelet involvement in COVID-19 thromboinflammation. Early detection and monitoring of COVID-19 increase the survival rate.

Thus, a continuous search for new biomarkers of severe acute respiratory syndrome coronavirus 2 infection is necessary for early diagnosis and stratification of COVID-19 severity to improve patient management. We hypothesized that circulating sCD40L and sCD62P levels are significantly modulated throughout the disease course of COVID-19 and significantly different from those in COVID-19 convalescent patients in the PLASMACOV cohort. We included 29 patients who attended the Pontchaillou University Hospital of Rennes, France, between March 2020 and July 2020. The patients with severe acute respiratory syndrome coronavirus 2 infection (with polymerase chain reaction positive tests) were hospitalized for severe COVID-19 in a continuing care or intensive care unit and received oxygen therapy (Table 1). Blood sampling was performed on intensive care unit admission (day 1) and days 3–5, 7, and 14. Plasma samples were obtained by centrifugation of citrated whole blood samples and stored at − 80 °C until assay. For the PLASMACOV cohort, in accordance with the recommendations of the European Blood Alliance, convalescent patients were considered eligible for plasma donation at least 14 days after symptom resolution and underwent the standard plasma apheresis procedure for healthy volunteers. Apheresis plasma samples from 26 convalescent patients were processed as usual with pathogen inactivation treatment and cryopreserved until clinical use. The sCD40L and sCD62P levels were measured using the Luminex technology or enzyme-linked immunosorbent assay (RnD Systems), respectively. Owing to some missing values due to the absence of blood samples for certain patients at some time points, a mixed model was used to evidence the significance of the changes of the sCD62P and sCD40L levels over time. The Tukey multiple-comparisons test was used to compare data from patients with COVID-19 with those from convalescent patients. Statistical differences were considered significant at *p* < 0.05.

**Table 1:** Patient characteristics and outcomes

	All patients (N = 29)	Convalescent patients (N = 26)
Age—years	57 (54–68)	37.6 (22–57)
*Sex*		
Female	8 (27.6%)	7 (26.9%)
Male	21 (72.4%)	19 (73.1%)
BMI—kg/m^2^	27.9 (24.4–31.5)	N/C
Days from illness onset to first blood sampling—days	11 (9–13)	N/A
SAPS II score on day 1	27 (16–35)	N/A
SOFA score on day 1	2 (1–8)	N/A
Platelet count on day 1–10^9^/L	219 (153–260)	N/A
Lymphocyte count on day 1–10^9^/L	0.8 (0.6–1.1)	N/A
Invasive ventilation	13 (44.8%)	N/A
ARDS	14 (48.3%)	N/A
Most pathological PaO2/FiO2	156 (89–238)	N/A
Acute renal failure	8 (27.6%)	N/A
Septic shock	2 (6.9%)	N/A
Thromboembolic event	4 (13.8%)	N/A
Hospital length of stay—days	11 (7–19)	N/A
ICU length of stay—days	4 (2–13)	N/A
Hospital death	1 (3.4%)	N/A

Results are presented as n (%) or median (IQR)

BMI, body mass index; SAPS II, Simplified Acute Physiology Score II; PaO2, arterial oxygen tension; FiO2, Fraction of Inspired Oxygen

N/C: Data not collected

N/A: Not applicable

**Fig. 1:**
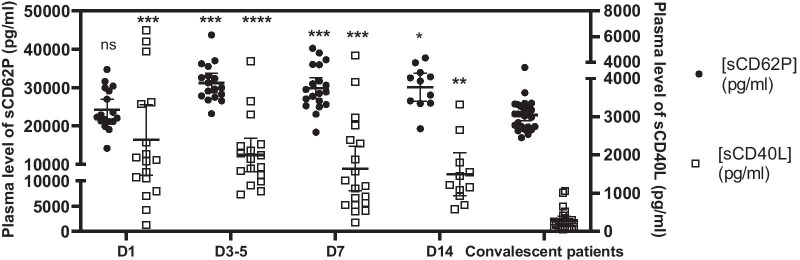
Plasma levels of sCD40L and sCD62P biomarkers in patients with severe COVID-19 throughout the disease course. Individual values and mean ± 95% confidence intervals are plotted for each sampling day (D1, D3–5, D7, and D14). A mixed model was used to evidence the significant changes in sCD62P and sCD40L levels in patients with COVID-19. The Tukey multiple-comparisons test was used to compare sCD62P and sCD40L values between COVID-19 and convalescent patients (*p < 0.05; **p < 0.005; ***p < 0.001; ****p < 0.0001)

Median age was 57 (54–68) years, 73% of the patients were men and the median BMI was 27.9 (24.4–31.5) (Table 1). The changes in the sCD40L and sCD62P concentrations are plotted in Fig. 1. The sCD62P level significantly increased over time from 24,251 to 33,784 pg/ml, whereas the sCD40L level decreased from 2396 to 1497 pg/ml (*p* < 0.0001). Moreover, the mixed model analysis revealed a significant interaction between the platelet inflammatory molecule and the time variables (*p* < 0.0001), indicating that the kinetics of the sCD40L plasma level significantly differed from that of sCD62P. Finally, the plasma levels of sCD62P and sCD40L of COVID-19 patients were significantly higher than those observed in the plasma samples of convalescent patients (22,886 and 290 pg/ml, for sCD62P and sCD40L respectively), regardless of sampling time, except for the sCD62P level assessed in the first time point, which did not differ significantly from those in convalescent patients. The strength of our study is the 14-day sequential sampling. Our data suggest that these factors are well associated with acute-phase COVID-19.

However, this study has several limitations. Indeed, the size of the population remains modest, even though we were able to perform several sequential samples on the same patients. Our observations need to be confirmed in a larger cohort of patients that would also allow to stratify COVID-19 patients according to clinical criteria, such as the most pathological PaO2/FiO2 or thromboembolic events.

On the other hand, it would be interesting to complete the kinetic analysis of the platelet inflammatory signature in COVID-19 patients by assessing other platelet factors. It would also be particularly relevant to combine the platelet inflammatory signature with other biological parameters, such as the percentage of circulating platelet-leukocyte complexes, in order to have a panel of biomarkers allowing to precisely characterize the thrombo-inflammatory state of COVID-19 patients.

## Conclusions

The management of COVID-19 infection remains complex partly because of the lack of reliable severity markers. Furthermore, our data highlight the difference in the temporal dynamics of these factors and the importance of monitoring relevant factors, which should include platelet factors, in the early stages of COVID-19 infection. Thus, the follow-up of platelet inflammatory parameters during the course of COVID-19 could be of particular interest for clinicians. Indeed, the assessment of the platelet signature of the thrombo-inflammation associated with severe COVID-19, particularly at early stages of the disease, will help patient monitoring, evaluation of the efficiency of therapeutic strategies on thrombo-inflammation and evidence the need for treatment adaptation, if levels of inflammatory factors are sustained.

Finally, our study suggest that platelets could be relevant therapeutic targets allowing clinicians to intervene early and simultaneously on two major systems, haemostasis and inflammation, that are profoundly deregulated during COVID-19.

## Data Availability

The datasets used and/or analyzed during the current study are available from the corresponding author upon individual specific and reasonable request.

## Abbreviations

SARS-CoV-2: Severe Acute Respiratory Syndrome Coronavirus-2
COVID-19: Coronavirus disease
(s)CD40L: Soluble (s)CD40L
(s)C62P: Soluble (s)CD62P
